# Surgical Responses of Medial Rectus Muscle Recession in Thyroid Eye Disease-Related Esotropia

**DOI:** 10.1371/journal.pone.0146779

**Published:** 2016-01-21

**Authors:** In Jeong Lyu, Ju-Yeun Lee, Mingui Kong, Kyung-Ah Park, Sei Yeul Oh

**Affiliations:** 1 Department of Ophthalmology, Samsung Medical Center, Sungkyunkwan University School of Medicine, Seoul, Korea; 2 Department of Ophthalmology, Eulji Medical Center, Eulji University School of Medicine, Seoul, Korea; University of Birmingham, UNITED KINGDOM

## Abstract

We evaluate the surgical outcomes and surgical responses of medial rectus muscle (MR) recession patients with thyroid eye disease (TED)-related esotropia (ET). The surgical dose-response curves 1 week postoperatively and at the final visit were analyzed. Univariable and multivariable linear regression analyses were applied to investigate factors influencing surgical dose-response. A total of 43 patients with TED-related ET that underwent MR recession were included. The final success rate was 86.0% and the rate of undercorrection was 14.0%. The surgical dose-response curves of TED-related ET showed a gentle slope compared with those of standard surgical tables. In the univariable model, simultaneous vertical rectus muscle recession was the only significant factor influencing surgical dose-response of MR recession in TED-related ET (β = -0.397, *P* = 0.044). In a model adjusted for age, sex, type of surgery, and preoperative horizontal angle of deviation, simultaneous vertical rectus muscle recession showed marginal significance (β = -0.389, *P* = 0.064). The surgical dose-response curve of TED-related ET was unique. Simultaneous vertical rectus muscle recession was associated with increased surgical dose-response in TED-related ET.

## Introduction

Thyroid eye disease (TED) is an autoimmune disorder characterized by inflammation and expansion of orbital fat and extraocular muscles (EOMs) [1]. Ophthalmic symptoms in TED vary and can include conjunctival injection, lid edema, eyelid retraction, proptosis, strabismus, and compressive optic neuropathy. TED is classified as a type I or type II disease based on the absence or presence of restrictive myopathy with diplopia within 20° of the primary position [2]. Type I disease is predominantly caused by orbital fat enlargement and accumulation of glycosaminoglycan and hyaluronic acid, which are produced by orbital fibroblasts. These patients show noninflammatory enlargement of retrobulbar fat with normal extraocular motility. In comparison, type II TED is associated with muscle enlargement and orbital inflammation, which result in limitation of extraocular motility and diplopia [2–5]. Impaired eye movement is observed in nearly 80% of patients with TED and, in 15 to 20% of cases, diplopia is the initial presentation [6–8].

Several reports investigated vertical muscle surgery in TED [9–15], because the inferior rectus muscle (IR) is most commonly involved in TED (60 to 80%) [7, 16]. However, only a few articles have evaluated horizontal muscle surgery, despite the medial rectus muscle (MR) being the second most frequently involved muscle (42 to 44%) in TED [7, 9, 16–18]. The purpose of this study was to evaluate the surgical outcomes and surgical responses of MR recession patients with TED-related esotropia (ET).

## Methods

This study was performed in accordance with the tenets of the Declaration of Helsinki. Approval to conduct this study was obtained from the Institutional Review Board of Samsung Medical Center.

### Patients

Retrospective chart review was performed for all patients who underwent unilateral (UMR) or bilateral MR recession (BMR) to treat TED-related ET between April 2003 and May 2013. All operations were performed by a single surgeon (SYO). Patients with restrictive strabismus other than TED, paralytic strabismus, highly myopic strabismus, sensory strabismus, neurological problems, < 3 months of follow-up, or a history of previous strabismus surgery were excluded.

Prism and alternate cover testing was performed at 6 m and 33 cm. Ocular ductions were graded from 0 to -4 with 0 indicating normal and -4 indicating severe limitation. Hertel exophthalmometry was examined and proptosis was defined as an exophthalmometry value of 17.0 mm or more [19]. Strabismus surgery was indicated when the ET was over 10 prism diopters (PD) at far and near distance, causing diplopia. Not only were patients euthyroid at the time of surgery, but their clinical activity scores for TED and strabismus were also stable for at least six months prior to surgical correction. The following data were collected from patient medical records: age at surgery, sex, manifested refraction, best corrected visual acuity (BCVA), the duration of thyroid disease and compressive optic neuropathy history, radiotherapy or systemic corticosteroid therapy, orbital decompression, exophthalmometry, the amount of deviation and ocular motility before and after strabismus surgery, type and magnitude of strabismus surgery, and the length of follow-up.

### Surgical procedure

The amount of MR recession was determined based on the distant angle of deviation measured at the final preoperative visit within one week of surgery. Patients with an esodeviation of less than 20 PD underwent UMR, and the rest underwent BMR based on the modified surgical dose-table in TED-related ET to prevent undercorrection based on the personal experience of the surgeon (Table 1). In cases of muscle adjustments, the procedure was performed 2 to 3 hours postoperatively when the patients were alert enough to cooperate with orthoptic measurements.

**Table 1:** Surgical tables of unilateral and bilateral medial rectus muscle recession in thyroid eye disease-related esotropia.

Preoperative deviation	Medial rectus muscle recession (mm)	
	Patients without TED	TED patients
15 PD	6.0*	6.5*
20 PD	7.0*	8.0†
25 PD	8.0†	9.0†
30 PD	9.0†	10.0†
35 PD	10.0†	11.0†
40 PD	11.0†	12.0†
50 PD	12.0†	13.0†
60 PD	13.0†	15.0†
70 PD	14.0†	17.0†

TED = thyroid eye disease

*Unilateral medical rectus muscle recession

†Total amount of bilateral medial rectus muscle recession in both eyes

### Postoperative measurements

Postoperative alignment at distance and near deviation was measured the first week, 1, 3, 6, 12 months and every year after surgery. Success was defined as ocular deviation within 10 PD at far and near distance. Undercorrection and overcorrection were defined as esodeviation of 10 PD or more and exodeviation of 10 PD or more, respectively. Change in deviation was determined by subtracting postoperative deviation at 1 week and at final visit from the preoperative deviation.

### Statistical analyses

Statistical analyses were performed using the SAS Enterprise Guide, version 4.3 (SAS Institute Inc, Cary, NC). Continuous variables were reported using median and interquartile range (IQR). Categorical variables were reported using number and proportion. Shapiro-Wilk test was used to assess the data for normality. The surgical dose-response curves 1 week postoperatively and at the final visit were analyzed using linear regression analysis. Univariable and multivariable linear regression analyses were also applied to investigate factors influencing surgical dose-response based on alignment at final visit. The multivariable model was adjusted for age, sex, type of surgery, and preoperative horizontal angle of deviation. *P* values less than 0.05 were considered statistically significant.

## Results

We identified a total of 43 patients with TED-related ET that underwent MR recession. All of the patients were Korean. The baseline characteristics were described in Table 2. The median age was 54 years (IQR 47–60) and 23 (53.5%) were female. The median duration of thyroid disease was 42 months (IQR 33–60). Twenty-eight patients (65.1%) underwent systemic steroid therapy, and 16 patients (37.2%) had a history of radiotherapy for active moderate-to-severe TED. Seven patients (16.3%) had a history of compressive optic neuropathy. Proptosis was shown in 14 patients (32.6%). The median exophthalmometric value was 15.0 mm (IQR 12.0–17.0). Eighteen patients (41.8%) underwent orbital decompression surgery, seven patients for compressive optic neuropathy and 11 patients for proptosis. All patients had BCVA ≥20/40 in both eyes. Among 43 TED-related ET patients, 32 patients (74.4%) underwent MR recession alone and 11 patients underwent MR recession with simultaneous vertical rectus muscle recession to correct vertical strabismus of 10 PD or more. Three patients underwent two vertical muscle recession procedures and the remaining patients underwent one vertical muscle recession. The median preoperative horizontal angle of deviation was 30 PD (IQR 20–50) and the median amount of MR recession was 11.5 mm (IQR 9.5–15.0). BMR was performed in 37 (86.1%) of patients and adjustable sutures were placed MR in 35 patients (81.4%). Among 35 patients with adjustable sutures, 12 (34.3%) required adjustment of the muscle position to achieve esodeviation within 4 PD at far and near without diplopia. Eleven patients (31.4%) exhibited an increased amount of recession and only one patient demonstrated recession reduced by 1.0 mm. The median amount of adjustment was 1.0 mm (IQR 0.5–1.25).

**Table 2:** Demographics of patients with thyroid eye disease-related esotropia (N = 43).

Variables	Median (IQR)
Age at surgery (years)	54 (47–60)
Female, n (%)	23 (53.5%)
Thyroid disease duration (months)	42 (33–60)
Previous orbital decompression surgery, n (%)	
Yes	18 (41.8%)
No	25 (58.2%)
History of systemic steroid therapy, n (%)	28 (65.1%)
History of radiotherapy, n (%)	16 (37.2%)
History of compressive optic neuropathy, n (%)	7 (16.3%)
Exophthalmometry value (mm)	15 (12–17)
Proptosis, n (%)	14 (32.6%)
Abductions of -3 and -4, n (%)	14 (32.6%)
Preoperative horizontal deviation (PD)	30 (20–50)
Amount of recession (mm) *	11.5 (9.5–15.0)
Simultaneous vertical rectus muscle recession, n (%)	
Yes	11 (25.6%)
No	32 (74.4%)
Type of surgery, n (%)	
UMR recession	6 (13.9%)
BMR recession	37 (86.1)
Adjustable sutures, n (%)	35 (81.4%)
Amount of suture adjustment (mm)	1.0 (0.5–1.25)
Postoperative follow-up time (months)	14.2 (5.2–31.7)

PD = prism diopters; UMR = unilateral medial rectus muscle; BMR = bilateral medial rectus muscle

*The total amount of medial rectus muscle recession in both eyes.

### Surgical outcome and surgical dose-response curves

The final success rate was 86.0% in TED-related esotropia and the rate of undercorrection was 14.0%. None of the patients had overcorrection after surgery.

**Fig 1:**
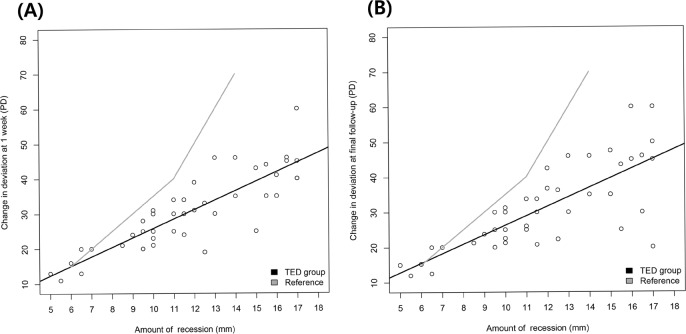
Surgical dose-response curves in TED-related esotropia. (A) Surgical dose-response curve based on alignment 1 week postoperatively. (B) Surgical dose-response curve based on alignment at final visit. The standard surgical table for esotropia was presented as a reference.

The surgical dose-response curves using linear regression analysis based on alignment 1 week postoperatively and at the final visit were shown in Fig 1. The linear regression equation based on the alignment 1 week postoperatively was: change in deviation = -1.15258 + 2.69163 * amount of recession. The linear regression equation based on the alignment at final visit was: change in deviation = -0.66098 + 2.69333 * amount of recession.

### Factors influencing surgical dose-response

Simple and multiple linear regression analyses were used to evaluate factors associated with final surgical response in TED-related ET (Table 3). In the univariable model, simultaneous vertical rectus muscle recession was the only significant factor influencing surgical dose-response of MR recession (β = -0.397, *P* = 0.044). In a model adjusted for age, sex, type of surgery, and preoperative horizontal angle of deviation, simultaneous vertical rectus muscle recession showed a marginally significant association with surgical dose-response (β = -0.389, *P* = 0.064). The median surgical response of 32 patients who underwent MR recession alone and 11 patients who underwent simultaneous vertical rectus muscle recession was 2.58 PD/mm (IQR 2.15–3.00) and 2.90 PD/mm (IQR 2.36–3.53), respectively.

**Table 3:** Factors influencing surgical dose-response in thyroid eye disease-related esotropia.

Variables	Unadjusted model			Adjusted model*		
	β	95% CI	p-value	β	95% CI	p-value
Age, per 10 year	0.001	-0.180, 0.181	0.995	-	-	-
Sex, female vs. male	-0.181	-0.543, 0.182	0.321	-	-	-
Preoperative horizontal angle of deviation	0.007	-0.004, 0.018	0.203	-	-	-
Unilateral vs. bilateral MR recession	0.261	-0.589, 0.467	0.817	-	-	-
Simultaneous vertical muscle surgery, yes vs. no	-0.397	-0.783, -0.011	**0.044**	-0.389	-0.801, 0.023	**0.064**
Exophthalmometry value	0.047	-0.060, 0.062	0.963	0.030	-0.051, 0.111	0.460
Abductions of -3 and -4, yes vs. no	0.144	-0.244, 0.532	0.459	0.290	-0.143, 0.722	0.183
Previous orbital decompression surgery, yes vs. no	0.230	-0.134, 0.594	0.210	0.260	-0.139, 0.659	0.195
History of systemic steroid therapy, yes vs. no	0.030	-0.342, 0.401	0.872	0.025	-0.369, 0.415	0.897
History of radiotherapy, yes vs. no	-0.166	-0.541, 0.209	0.378	-0.128	-0.538, 0.283	0.533
History of compressive optic neuropathy, yes vs. no	0.065	-0.430, 0.561	0.791	0.102	-0.437, 0.640	0.704

CI = confidence interval

Adjusted for age, sex, preoperative horizontal angle of deviation, and unilateral vs. bilateral surgery

## Discussion

Many previous reports recommend performing strabismus surgery when patients are euthyroid and exhibit stable orthoptic measurements for at least six months [18, 20–23]. Even under these conditions, success rates vary greatly, ranging from 55 to 100%, and undercorrection rates are relatively high [10, 18, 20–31]. It can be challenging to select the appropriate amount of surgical intervention needed in TED patients with strabismus. One previous study reported that there was lesser surgical response compared to the expected surgical response based on a standard surgical table in patients with TED-related esotropia [16]. Another study that suggested a new surgical technique for TED-related strabismus found that the correlation between preoperative deviation and the amount of MR recession did not fit the standard surgical nomogram in TED [18].

In this study, 43 patients with TED-related ET that underwent MR recession were evaluated for surgical outcomes and surgical responses using an augmented surgical table. The success rate was 86.0% and the undercorrection rate was 14.0%. None of the patients showed overcorrection. A large amount of recession was needed in TED-related ET patients. Moreover, among the 35 patients who underwent the adjustable suture technique, 11 patients (31.4%) needed further recession even after use of the augmented surgical tables. The surgical dose-response curves in TED-related ET showed a gentle slope compared with standard surgical tables, which indicated reduced surgical dose-response. This agrees with a previous study by Mocan et al [16]. The reduced surgical dose-responses of MR recession in TED-related ET could be related to pathologic changes in the EOMs and other periorbital tissues that are not present in patients without underlying TED. The fibrotic changes and MR muscle thickening that occur in TED are resistant to the recession procedure [32]. Other structural changes in the orbit also might affect surgical response. A crowded orbit caused by proliferation of orbital fat, infiltration of mucopolysaccharide ground substance and bulky EOMs may provide insufficient space for recession of the MR. In comparison, orbital decompression can alter the orbital structure and may influence not only developing strabismus, but also surgical response. Another possible explanation is that pathologic changes in vertical rectus muscles in TED could play a role in the reduced response to MR recession, since adduction is the secondary action of the vertical rectus muscles [33, 34]. Therefore, we further investigated factors influencing surgical dose-response in TED-related ET including simultaneous vertical rectus muscle, exophthalmometry, limitation of abduction, history of radiotherapy, and previous orbital decompression. Our results showed that simultaneous vertical rectus muscle recession was associated with increased surgical dose-response in TED-related ET, although it was marginally significant (*P* = 0.064) after adjustment for age, sex, type of surgery, and preoperative horizontal deviation. Patients who underwent simultaneous vertical rectus muscle recession showed greater surgical response than did those with MR recession only. The effects of MR recession could be restricted by enlarged vertical rectus muscles and could be improved by simultaneous vertical rectus muscle recession. However, contrary to our results, a previous report suggested that the effect of concomitant IR recession on horizontal deviation was negligible, although a mild increase in surgical response was found in patients who underwent concomitant IR recession [16]. This disparity may be due to differences in the proportion of TED-related ET patients who do and do not undergo vertical rectus muscle surgery. Other considerations that may explain the variation in results include differences in the severity of pathologic changes in vertical rectus muscles, preoperative vertical misalignment, and the amount of vertical rectus muscle recession between two studies. Prospective studies with a large sample size are needed to determine the effect of simultaneous vertical rectus muscle recession on MR recession in TED-related ET.

Previous studies regarding the effect of orbital decompression on TED-related strabismus yielded controversial results. A study by Mocan et al. demonstrated that a history of decompression was associated with reduced surgical response and could result in less favorable outcomes [16]. On the other hand, a study by Dal Canto et al. found that previous orbital decompression had no effect on reoperation rate, although more muscles required surgery in patients who had undergone decompression [18]. A previous report by our group also indicated that the surgical success rate was not different in groups who did and did not undergo orbital decompression [31]. Similarly, the present study showed that there was no significant association between previous orbital decompression and surgical dose-response of MR recession. Differences in orbital decompression surgical techniques, the reason for orbital decompression, and ethnicity might explain this disparity. Thus, further prospective studies are needed to confirm our findings.

TED is common in females; the female-to-male ratio is 4:1 [3]. However, a more equal gender distribution is observed in cases of severe ophthalmopathy [3, 16]. In the present study, the female-to-male ratio of TED-related ET was 23:20, while 63% of patients were female in a previous study of TED-related ET [16]. The large number of cases with severe TED included our study may have contributed to this finding.

Another remarkable finding was that the postoperative angle of deviation was altered in TED-related esotropia, especially in patients with large amounts of recession (over 15 mm). Compared with the results of 1 week postoperatively, a scatter plot of the final visit results showed considerable variation in the regression line. The postoperative course of strabismus related to TED is less predictable because of the underlying disease process and the final outcome is not necessarily guaranteed, even when eyes show good alignment early in the postoperative period.

There were several limitations to our study. The study was retrospective in design and follow-up intervals were not standardized. Secondly, other potential factors such as the size of the EOMs were not analyzed in this study. Further studies evaluating the relationship between surgical dose-response and the size of the EOMs would help us better understand the poor surgical response of MR recession in TED.

In conclusion, the surgical dose-response curve of TED-related ET was unique. Simultaneous vertical rectus muscle recession was associated with increased surgical dose-response in TED-related ET.
